# AM-18002, a derivative of natural anmindenol A, enhances radiosensitivity in mouse breast cancer cells

**DOI:** 10.1371/journal.pone.0296989

**Published:** 2024-04-16

**Authors:** Da-Young Eum, Myeonggyo Jeong, Soon-Yong Park, Jisu Kim, Yunho Jin, Jeyun Jo, Jae-Woong Shim, Seoung Rak Lee, Seong-Joon Park, Kyu Heo, Hwayoung Yun, Yoo-Jin Choi

**Affiliations:** 1 Research Center, Dongnam Institute of Radiological & Medical Sciences, Busan, Republic of Korea; 2 College of Pharmacy, Pusan National University, Busan, Republic of Korea; 3 Research Institute for Drug Development, Pusan National University, Busan, Republic of Korea; Abu Dhabi University, UNITED ARAB EMIRATES

## Abstract

Natural anmindenol A isolated from the marine-derived bacteria *Streptomyces* sp. caused potent inhibition of inducible nitric oxide synthase without any significant cytotoxicity. This compound consists of a structurally unique 3,10-dialkylbenzofulvene skeleton. We previously synthesized and screened the novel derivatives of anmindenol A and identified AM-18002, an anmindenol A derivative, as a promising anticancer agent. The combination of AM-18002 and ionizing radiation (IR) improved anticancer effects, which were exerted by promoting apoptosis and inhibiting the proliferation of FM3A mouse breast cancer cells. AM-18002 increased the production of reactive oxygen species (ROS) and was more effective in inducing DNA damage. AM-18002 treatment was found to inhibit the expansion of myeloid-derived suppressor cells (MDSC), cancer cell migration and invasion, and STAT3 phosphorylation. The AM-18002 and IR combination synergistically induced cancer cell death, and AM-18002 acted as a potent anticancer agent by increasing ROS generation and blocking MDSC-mediated STAT3 activation in breast cancer cells.

## Introduction

Cancer remains the leading cause of human death worldwide and is mainly characterized by uncontrolled and irregular cell proliferation and growth [1]. Radiation therapy (RT), along with chemotherapy and surgery, is a mainstream cancer treatment method [2–4]. In RT, ionizing radiation (IR) is applied to kill cancer cells and prevent tumor progression and recurrence [4]. However, tumor radioresistance causes RT failure and consequent tumor relapse, especially in patients with solid tumors. A possible solution for radioresistance is to increase local energy deposition at the tumor while ensuring space surrounding healthy tissues. Radiosensitizers [5], which are agents sensitizing tumor cells to radiation, can offer this solution [6]. Therefore, effective radiosensitizers that can overcome radioresistance must be investigated to improve cancer treatment effectiveness.

Mitochondria are organelles essential for cell survival, death, and signaling and are among the primary sites of production of reactive oxygen species (ROS) [7, 8]. These organelles also play a prominent regulatory role in apoptosis [9–11]. ROS levels are typically higher in tumor cells and the tumor environment (TME), and serve as effector molecules of radiation, contributing to radiation-induced DNA damage and cancer cell death [12, 13].

The TME is a crucial player in tumor progression, treatment responses, and patient prognosis. Myeloid-derived suppressor cells (MDSCs) are a heterogeneous population of myeloid progenitor cells in the inflammation-, tumor progression-, and metastasis-associated TME [14, 15]. The signal transducer and activator of transcription 3 (STAT3) is an oncogenic protein, and the STAT3 signaling pathway has a critical role in tumor cell occurrence and development [16]. Treatment with STAT3 inhibitors alone or in combination with other clinical therapeutic drugs may have more promising effects on suppressing or reversing chemoresistance in breast cancer [17]. Yin et al. recently reported that an Aurora-A kinase inhibitor (Alisertib) is involved in regulating the immunosuppressive functions of STAT3 and MDSCs in breast cancer TME [18]. Overall, STAT3-related MDSC generation is a major obstacle to antitumor immunotherapy.

This study investigated whether the AM-18002 and RT combination synergistically induced cancer cell death and whether AM-18002 exerted its anticancer effect by inhibiting STAT3-related MDSC generation in FM3A breast cancer cells *in vitro*.

## Materials and methods

### Preparation of AM-18002

AM-18002 was synthesized according to our previous procedures [19]. Its structure was confirmed through nuclear magnetic resonance spectroscopy, liquid chromatography-mass spectrometry, and infrared spectroscopy. For the *in vitro* experiments, AM-18002 was dissolved in dimethyl sulfoxide (DMSO) (#D2650, Sigma-Aldrich) to prepare a 10 mM stock solution and stored at -20°C.

### Determining the purity of AM-18002

A 10 mM stock solution of AM-18002 in DMSO was used for analysis. This solution was filtered through a 0.45-mm hydrophobic PTFE filter and analyzed through semi-preparative HPLC by using SPD-20A/20AV Series Prominence HPLC UV-Vis detectors (Shimadzu, Tokyo, Japan). The analysis was performed by injecting 10 μL of the sample into a Phenomenex Luna C18 (4.6 × 100 mm, 3.5 μm) column. For the post-run reconditioning of the column, the mobile phase consisting of formic acid in H_2_O [0.1% (v/v)] (A) and acetonitrile [0.1% (v/v)] (B) was delivered at a 2 mL/min flow rate by applying the following programmed gradient elution: 0%–100% (B) for 50 min, 100% (B) for 1 min, 100% (B) isocratic for 10 min, and then 0% (B) isocratic for 10 min. AM-18002 was detected at 23.30 min (retention time) through semi-preparative HPLC analysis.

### *In silico* drug-likeness and ADME profile prediction

The Swiss ADME online software is used to evaluate the drug-likeness and pharmacokinetic of chemical compounds before the molecular modeling study [20]. The ADME feature of the molecule was evaluated and analyzed using the online system pkCSM (https://biosig.lab.uq.edu.au/pkcsm/) [21].

### Molecular docking analysis

To investigate the binding propensities of AM-18002 to the SH2 domain of STAT3, a docking simulation was performed as described elsewhere [22]. The 3D structure of the ligand was prepared and minimized by MM2 using Chem 3D pro 15.1 software. The STAT3 structure was downloaded from the RCSB protein data bank (PDB code: 1BG1). Using UCSF chimera 1.15, chain A of the receptor was prepared for docking simulation by removing the other chain and all non-standard residues. The ligand and protein files were processed using the AutoDock Protocol [23]. Docking simulation was performed using a Lamarckian Genetic Algorithm. The hydrogen binding analysis and visual investigations with the ligand and receptor were conducted using UCSF chimera 1.15 [24].

### Cell line and culture

The mouse mammary carcinoma cell line FM3A (RRID:CVCL_3869) was cultured every 2 days in RPMI 1640 medium (#10-040-CV, Corning) containing 10% fetal bovine serum (FBS) (#35-015-CV, Corning) and 1% antibiotic-antimycotic solution (#15240–062, Gibco). The FM3A cells were maintained in a humidified incubator under a 5% CO_2_ atmosphere at 37°C.

### Animals

C3H/He female mice (age: 5 weeks) were purchased from Central Lab Animal Incorporation (Seoul, Korea) and were maintained under specific pathogen-free (SPF) conditions. The FM3A cells (2 × 10^6^ cells/50 μL) were subcutaneously injected into the right flanks of the CH3/He mice. After approximately 4 weeks, the mice were euthanized through carbon dioxide inhalation. Spleen cells were isolated from the tumor-bearing mice, and MDSCs were isolated from their spleen cells by using the magnetic-activated cell sorting (MACS) method. Animal experiments were performed according to the protocols approved by the Institutional Animal Care and Use Committee of the Dongnam Institute of Radiological & Medical Sciences (DIRMAS) (Busan, Korea) (Approval No. DIRMAS AEC-2021-009).

### Cell sorting and flow cytometry analyses

Spleen cells isolated from the FM3A cell tumor-bearing mice were depleted of red blood cells by using ACK Lysing Buffer (#A1049201, Gibco, Thermo Fisher Scientific) and washed twice with cold PBS. The spleen cells were then separated by labeling them with the MDSC marker mouse anti-Ly-6G UltraPure MicroBeads (#130-120-337, Miltenyi Biotec) for 10 min at 4°C. The cells were then sorted through the magnetic-activated cell sorting (MACS) method by using the LS column (#130-042-401, Miltenyi Biotec) containing the MACS stock solution (#130-091-376, Miltenyi Biotec). To analyze the purity of the cell population, the cells were resuspended in 100 μL of 1% FBS solution in PBS and incubated with anti-CD11b [fluorescein isothiocyanate (FITC)-conjugated] (#130-113-805, Miltenyi Biotec) and anti-Gr1 (APC-conjugated) (#130-112-149, Miltenyi Biotec) for 20 min at 4°C. The cell pellets were resuspended in 400 μL of 1% FBS solution in PBS and analyzed through flow cytometry (Navios Analyzer, Beckman Coulter, Inc.). MDSCs were analyzed using FLowJo software.

### Co-culture

The FM3A cells (1 × 10^5^/2 mL) were seeded into each well of a 6-well plate (#353046, Falcon), and the spleen cells or MDSCs (1×10^6^/3 mL) were seeded into the insert of a 6-well plate with a 1.0-μm pore size (#353102, Falcon). The spleen cells or MDSCs were treated with different AM-18002 concentrations (0, 1.5, 3, and 6 μM). All cell types were co-cultured for 72 h and then harvested. These cells were analyzed through flow cytometry (Navios Analyzer, Beckman Coulter, Inc.) and subjected to invasion and migration assays and western blotting.

### Viability test

The FM3A cells (1 × 10^5^ cells/well) were seeded into 6-well plates, cultured for 24 h, and treated with 0, 3, 6, 12.5, 25, and 50 μM AM-18002 for 24, 48, and 72 h to establish an adequate AM-18002 regimen without overt toxicity. Moreover, MDSCs were seeded into 6-well plates, cultured for 24 h, and treated with 0, 3, 6, 12.5, 25, and 50 μM of AM-18002 for 72 h. Subsequently, 500 μL MTT reagent (Beyotime Biotechnology) was added to each well and incubated for another 4 h at 37°C. Finally, the culture medium was replaced with 500 μL DMSO. To assess cell viability, optical density was measured at 490 nm by using a SpectraMax Paradigm microplate spectrophotometer (Molecular Devices).

### Clonogenic cell survival assay

The FM3A cells were pretreated with different concentrations (0, 12.5, and 25 μM) of AM-18002. The next day, the cells were harvested and seeded into 100-mm culture plates at a density of 1,000 cells/mL. After 2 h, the cells were exposed to gamma rays from a ^137^Cs gamma-ray source (Eckwert & Ziegler) at a dose rate of 2.6 Gy/min. After the cells were subjected to different doses (0, 0.5, 1, 2, 4, and 6 Gy) of IR, the cells were incubated for 10 days. The colonies formed were washed with PBS, fixed with methanol for 10 min, stained with 0.5% crystal violet for 2 h, and washed with distilled water. After cell staining, the colonies containing more than 50 cells were counted and survival curves were generated.

### Annexin V/propidium iodide staining

The FM3A cells (1×10^5^ cells/well) were seeded in 6-well plates and cultured for 24h. The cells were pretreated with different concentrations (0, 12.5, and 25 μM) of AM-18002 for 2 h, and exposed to IR at 0, 4, and 8 Gy. The next day, the cells were harvested and washed with PBS. The pellets were resuspended in 1× Annexin V binding buffer (#556547, BD Biosciences) at a concentration of 1×10^6^ cells/mL. Next, 100 μL of the solution was transferred to a 5 mL culture tube, and 5 μL of FITC Annexin V and 5 μL of propidium iodide (PI) were added to the tube. The cells were then gently vortexed and incubated for 15 min at room temperature in the dark. Next, 400 μL of 1× binding buffer was added to each tube, and the samples were analyzed through flow cytometry (FACSAria cell sorter, BD Biosciences) within 1 h. The percentages of cells within a population that are actively undergoing apoptosis were analyzed using FlowJo software.

### JC-10 mitochondrial membrane potential assay

Changes in the mitochondrial membrane potential were assessed in the FM3A cells at 24 h after the AM-18002 and IR combination treatment by using the JC-10 Mitochondrial Membrane Potential Assay Kit (#ab112133, Abcam). The FM3A cells were pretreated with different concentrations (0, 12.5, and 25 μM) of AM-18002 for 2 h and then exposed to IR at 0, 4, and 8 Gy. The next day, the cells were collected, washed with 1% FBS in PBS, stained for 30 min in the dark at room temperature, and analyzed through flow cytometry (Navios Analyzer, Beckman Coulter, Inc.). The percentages of green (apoptotic/necrotic) and orange/red (normal) cells were analyzed through FlowJo software.

### Western blotting

The FM3A cells were pretreated with different concentrations (0, 12.5, and 25 μM) of AM-18002 for 2 h and then exposed to IR at 0, 4, and 8 Gy. The next day, the cells were washed with cold PBS and lysed using lysis buffer (#TLP-121CETi, TransLab). The protein concentration was quantified using the Bio-Rad Protein Assay Kit (#5000001, Bio-Rad). Protein samples (10 μg) were loaded onto 4%–12% Bis-Tris Plus gradient gels (#NW04120, Invitrogen) and then transferred to polyvinylidene fluoride membranes (#10600100, GE Healthcare). The membranes were blocked with 5% skim milk (#232100, BD) at room temperature for 1 h and incubated with primary antibodies at 4°C overnight. Following incubation, the membranes were washed three times with Tris-buffered saline with Tween20 (TBS-T) and reprobed with secondary antibodies at room temperature for 2 h. Finally, the membranes were developed using ECL solution (#RPN2109, GE Healthcare) and visualized and captured using an Amersham ImageQuant 800 (Amersham) imaging system.

### Antibodies

Western blotting was performed using cleaved caspase-9 (Asp353) (#9509, Cell Signaling), cleaved caspase-3 (Asp175) (#9661, Cell Signaling), PARP (#9542, Cell Signaling), p-Stat3 (Tyr705) (#9145, Cell Signaling), Stat3 (#9139, Cell Signaling), and ß-actin (#SC-47778, Santa Cruz) antibodies.

### ROS generation

ROS levels in the FM3A cells treated with different AM-18002 concentrations and IR were measured using a commercial kit (DCFDA/H2DCFCA-Cellular ROS Assay Kit, #ab113851, Abcam) according to the manufacturer’s protocols. The cells were pretreated with different concentrations (0, 12.5, and 25 μM) of AM-18002 for 2 h and then exposed to IR at 0, 4, and 8 Gy. The next day, the cells were collected, washed with 1% FBS in PBS, stained for 30 min in the dark at room temperature, and analyzed through flow cytometry (Navios analyzer, Beckman Coulter, Inc.). The ROS levels were analyzed using FlowJo software.

### γ-H2AX foci staining

The FM3A cells were plated on poly-L-lysine-coated glass coverslips (#72292, EMS) in 6-well plates. The next day, the cells were pretreated with different concentrations (0, 12.5, and 25 μM) of AM-18002 for 2 h and then exposed to IR at 0, 4, and 8 Gy. After 24 h, the cells were fixed with 100% methanol for 5 min, permeabilized with 0.1% PBS-Triton X-100 for 5 min, blocked with 1% BSA in 0.1% PBS-Tween20 for 1 h, and incubated with anti-gamma H2A.X (phosphor S139) primary antibody (#ab11174, Abcam, 1:100) at 4°C overnight. The cells were then incubated with goat antirabbit IgG H&L (Alexa Fluor 488) preabsorbed (#ab15008, Abcam, 1:200) secondary antibody at room temperature for 2 h. The slides were washed, dried, and mounted (#H-1400-10, VECTASHIELD HardSet Antifade Mounting Medium). γ-H2AX staining was performed according to the Abcam immunofluorescence staining protocol. Images were captured using a Nikon Eclipse NI-E fluorescence microscope (Nikon). All images were analyzed using NIS-Elements BR analysis software.

### Comet assay

The FM3A cells were pretreated with different concentrations (0, 12.5, and 25 μM) of AM-18002 for 2 h and then exposed to IR at 0, 4, and 8 Gy. The next day, the cells were mixed with low-melting agarose at a 1:10 volume ratio and smeared on the slides. The slides were placed at 4°C for 30 min in the dark, immersed in lysis solution at 4°C overnight, and treated with an alkaline unwinding solution to relax and denature DNA at 4°C for 1 h. The samples were electrophoresed in an electrophoresis system tank (#4250-050-ES, R&D Systems) with precooled alkaline electrophoresis solution at pH >13 and 21 V for 40 min and then stained with SYBR Gold Nucleic Acid Gel Stain (#S11494, Invitrogen) for 30 min in the dark. The comet assay was performed using the Comet Assay Kit (#4250-050-K, Trevigen). Images were captured under a Nikon Eclipse NI-E fluorescence microscope (Nikon) and analyzed using NIS-Elements BR analysis software.

### Cell migration and invasion assay

First, 8-μm pore size Transwell inserts (#353097, Falcon) were placed in 24-well plates and coated with the Matrigel matrix (Matrigel Basement Membrane Matrix, Corning) for 1 h. The FM3A cells co-cultured with MDSCs at different concentration (0, 1.5, 3, and 6 μM) for 72 h were harvested. In the upper compartment, 150 μL of FM3A cells (1 × 10^5^ cells/mL) was gently added to a serum-free medium. Then, 800 μL of culture medium was added to the lower compartment. The inserts were incubated overnight and then carefully removed. To fix the cells on the lower side of the insert filter, 100% methanol was used for 10 min and stained with 0.5% crystal violet solution for 1 h. After the washes, the insert membrane was thoroughly dried. The number of cells was counted under an EVOS microscope (EVOS XL Cell Imaging System, Life Technologies) at 200× magnification.

### Statistical analysis

The data were analyzed and plotted using GraphPad Prism 8. The results are expressed as the mean ± standard deviation (SD). Significant differences between the treatments and control were evaluated through an analysis of variance (Dunnett’s test, PASW Statistics 18). P < 0.05 indicated statistical significance.

## Results

### *In silico* drug-likeness and ADME profile prediction

The calculated scores suggested that AM-18002 exhibits favorable drug-likeness (Table 1). AM-18002 satisfied the criteria of Topological Polar Surface Area (≤140 Å), with an Absolute Solubility Score of 0.55, which indicated its high potential for oral bioavailability [25–27]. The investigated compound demonstrated reasonable physicochemical properties, including ClogP and LogS values within the lead-likeness range [28]. Moreover, it fell under the moderately soluble category based on the Ali solubility class. Additionally, AM-18002 is predicted to exhibit a high likelihood of gastrointestinal absorption, but it is not expected to be a substrate for p-glycoprotein. Finally, AM-18002 was assessed for drug-likeness and the absence of Pan Assay Interference Structures [29, 30]. No structural alerts were identified.

**Table 1:** Drug-likeness prediction for AM-18002.

Comp.	TPSA (Å^2^)	ABS	CLogP	Ali LogS	Ali class	GA	Pgp	Drug-likeness (# viol.)	PAINS
AM-18002	20.23	0.55	3.10	-2.58	MS	High	No	Yes (0)	0 alerts

AM-18002 exhibits a Log S value of −3.702, which indicates favorable water solubility among the listed ADME features. According to the pkCSM web server, the standard range for good Caco-2 permeability is >0.90 [21]. We noted that AM-18002 surpasses this standard, exhibiting excellent Caco-2 permeability (Table 2). The distributional features of AM-18002, including volume distribution (VD) and blood–brain barrier (BBB) permeability, are also presented in Table 2. A lower VD suggests a more significant plasm drug concentration, which indicates less effective tissue distribution [31]. AM-18002, in this context, exhibited low VD levels and BBB permeability. However, AM-18002 may be inhibited by CYP450 1A2 in the metabolic aspect. The total clearance of AM-18002 is approximately 0.209 (mL/min/kg), suggesting that the body can eliminate a maximum of 0.209 (mL/min/kg) of the drug. Moreover, AM-18002 is not excreted through the renal OCT2 substrate. In conclusion, this computational ADME study provides evidence that AM-18002 has favorable biological characteristics as a potential drug candidate.

**Table 2:** ADME profiling prediction for AM-18002.

Comp.	Absorption		Distribution		Metabolism		Excretion	
	Water solubility Log S	Caco-2 Permeability 10^−6^ cm/s	VDss (human)	BBB permeability	CYP450 1A2 Inhibitor	CYP450 2D6 substrate	Total clearance (ml/min/kg)	Renal OCT2 substrate
AM-18002	-3.702	1.466	0.832	0.371	YES	NO	0.209	NO

### Viability of AM-18002-treated cells

**Fig 1:**
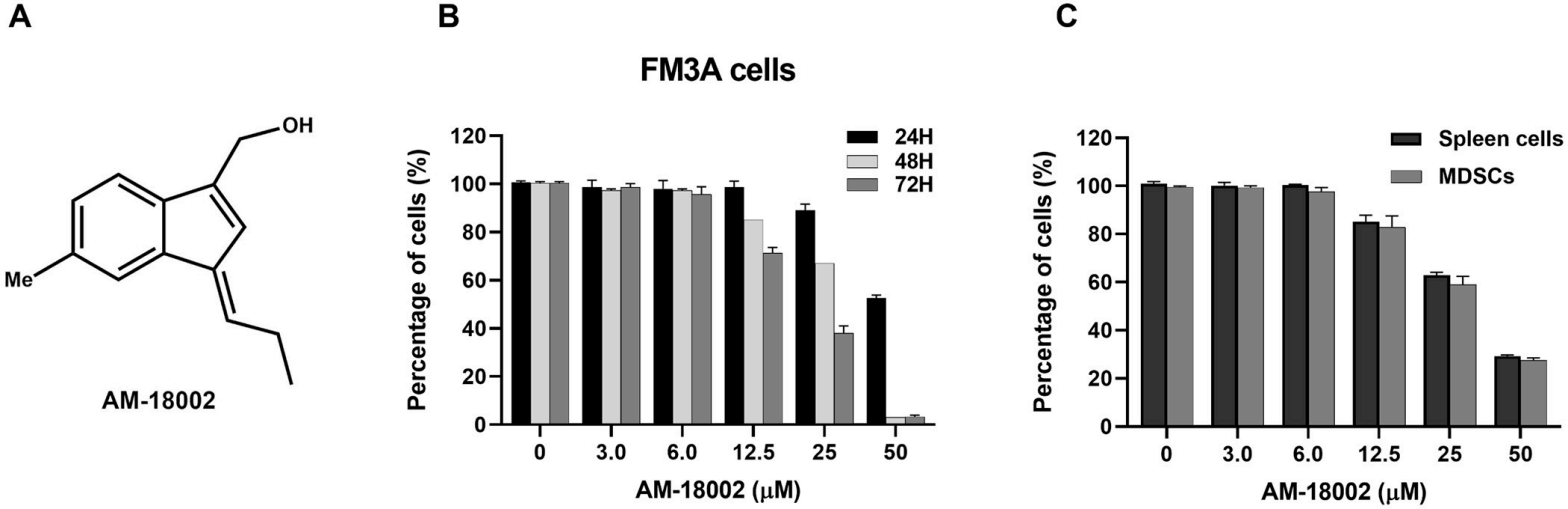
Effect of AM-18002 on FM3A cells and MDSCs viability. (A) The chemical structure of AM-18002. (B) FM3A cells and (C) MDSCs were treated with AM-18002 at different concentrations (0, 3, 6, 12.5, 25, and 50 μM) for different durations (24, 48, and 72 h). Cell viability was measured after incubation by using the MTT assay.

**Fig 2:**
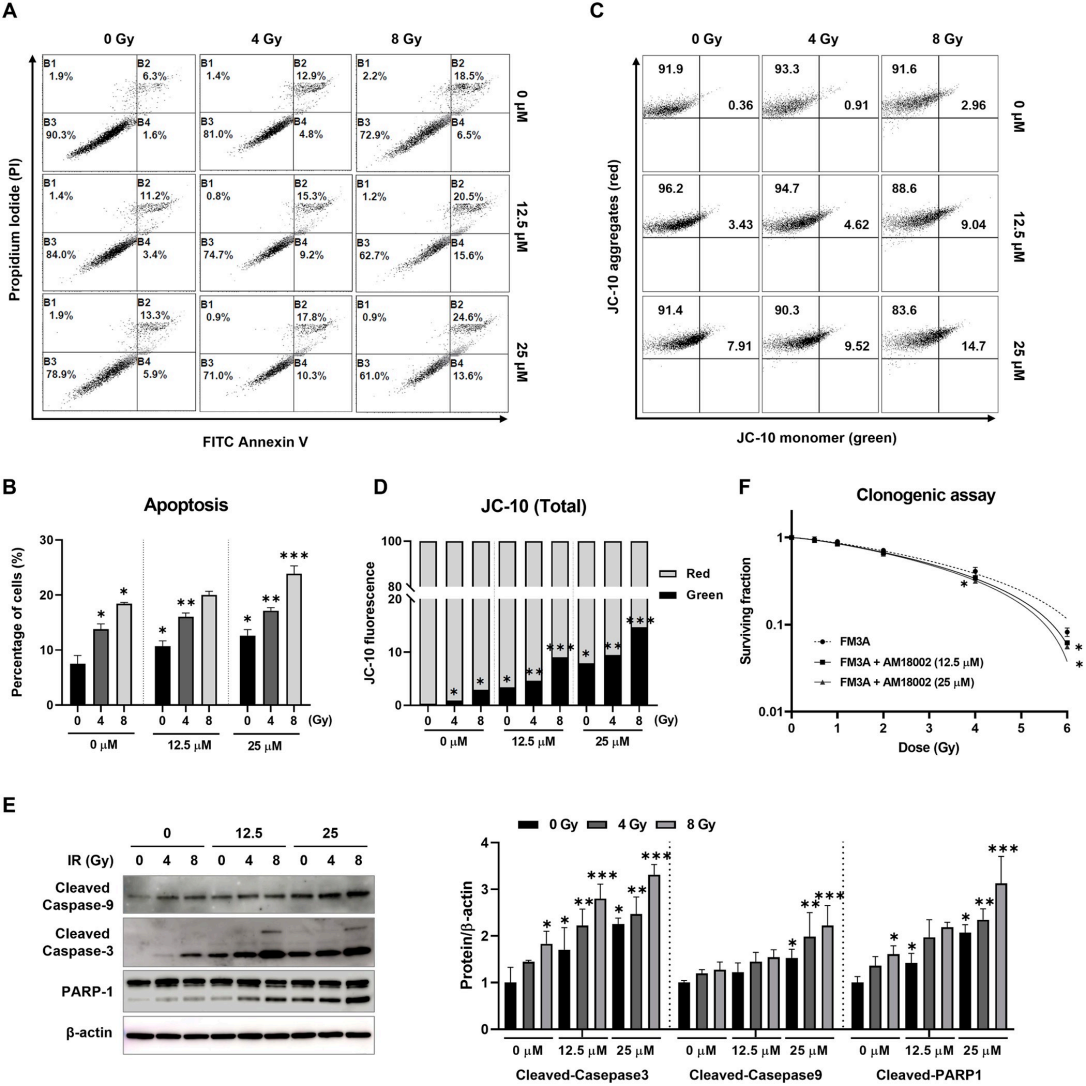
AM-18002 induces mitochondria-mediated apoptosis in irradiated FM3A cells. FM3A cells were untreated, treated with ionizing radiation (4 or 8 Gy), or treated with IR combined with AM-18002 for 24 h. (A) The cells were incubated with both annexin V and PI and analyzed by flow cytometry. The viable cells were not stained by Annexin V and PI, as shown in the lower left quadrant. (B) Apoptotic and/or necrotic cells in the upper right quadrant were stained with both Annexin V and PI. The quantitation of cell death (%) by apoptosis and/or necrosis. (C) The effect of cotreatment with AM-18002 and irradiation on mitochondrial transmembrane permeability transition. The mitochondrial membrane potential (MMP, ΔΨm) was assessed by JC-10 fluorescence by flow cytometry. (D) The percentage of MMP loss expressed as JC-10 green-positive cells. (E) Cleaved caspase-9/-3 and PARP-1 levels were verified by Western blotting. ß-actin was used as the loading control. (F) FM3A cells were pretreated with AM-18002 (12.5 and 25 μM) for 24 h and exposed to varying radiation doses. Clonogenic survival assays were performed in 10 cm dishes cultured for up to 10 days after radiation or combined treatment. Radiation only (●), AM-18002 plus radiation (■ and ▲). *P < 0.05 indicates a significant difference compared to the respective radiation-only group. The results are presented as the mean ± SD. *P < 0.05 vs. 0 Gy (0 μM), **P < 0.05 vs. 4 Gy (0 μM), ***P < 0.05 vs. 8 Gy (0 μM).

The effects of AM-18002 on the viability of FM3A mouse breast cancer cells and spleen cells were determined through the MTT assay. The spleen cells were isolated from the FM3A cell tumor-bearing mice after removing the red blood cells. No marked reduction in cell viability was observed at 24 h in the FM3A cells treated with ≤12.5 μM AM-18002, whereas treatment with 25 μM AM-18002 was cytotoxic (Fig 1B). Therefore, we used 12.5 and 25 μM AM-18002 in the subsequent experiments. MDSCs and spleen cells with 0, 3, and 6 μM AM-18002 exhibited no obvious cytotoxicity at 72 h (Fig 2C). Thus, 6 μM was selected as the upper dose limit in this study.

### AM-18002 significantly enhances the apoptosis of irradiated FM3A cells

Apoptosis is a major form of chemotherapeutic agent-induced cell death and is implicated in the cytotoxic mechanisms of various chemotherapy types [32]. We first determined whether AM-18002 increased FM3A cell radiosensitivity by inducing apoptosis and compared the effects of AM-18002 combined with IR through flow cytometry by using the Annexin V-FITC/PI kit. Annexin V-FITC and PI dual-staining unveiled that treatment with 12.5 or 25 μM AM-18002 plus IR synergistically induced apoptosis (Fig 2A and 2B). A JC-10 assay and flow cytometry were performed to investigate the effects of AM-18002 on the mitochondrial membrane potential. The FM3A cells treated with the AM-18002 and IR combination for 24 h exhibited a greater loss of membrane potential than those treated with irradiation alone (Fig 2C and 2D). Altogether, the aforementioned results indicated that AM-18002 increases the radiosensitivity of the FM3A cells, at least partially by inducing apoptosis.

Next, the expression of proteins associated with mitochondria-mediated apoptosis was investigated to identify signaling pathways associated with AM-18002-enhanced radiation-induced apoptosis. Caspase family members are critical regulators of cell apoptosis [33]. Irradiation dose-dependently upregulated cleaved caspase-9 and 3. Of note, western blotting revealed that the upregulation of these proteins in the cells treated with the AM-18002 and IR combination was greater than that in the cells treated with irradiation alone. Cleaved PARP, one of the most pivotal biochemical markers of caspase-3 activation-triggered cell apoptosis, was also upregulated by the combined treatment of AM-18002 and IR compared with the irradiation alone treatment, as exhibited by western blotting (Fig 2E), which confirmed that AM-18002 exerted a radiosensitizing effect on FM3A cells by enhancing IR-induced apoptosis. Together, these results indicated that the caspase-3 pathway was involved in AM-18002-induced apoptosis of irradiated FM3A cells.

We further investigated the effect of AM-18002 on FM3A cell radiosensitivity through the clonogenic assay. The cells were pretreated with AM-18002 for 24 h, irradiated with 0, 0.5, 1, 2, 4, and 6 Gy, and cultured for 10 days. Irradiation significantly dose-dependently decreased the colony formation of cancer cells. The number of AM-18002-treated colonies after irradiation significantly reduced compared with the number of cells treated with irradiation alone (Fig 2F). These results demonstrated that AM-18002 significantly reduces the ability of dying cells to repair IR-induced damage.

### AM-18002 induces ROS production and modulates the abundance of DNA double-strand breaks in irradiated FM3A cells

**Fig 3:**
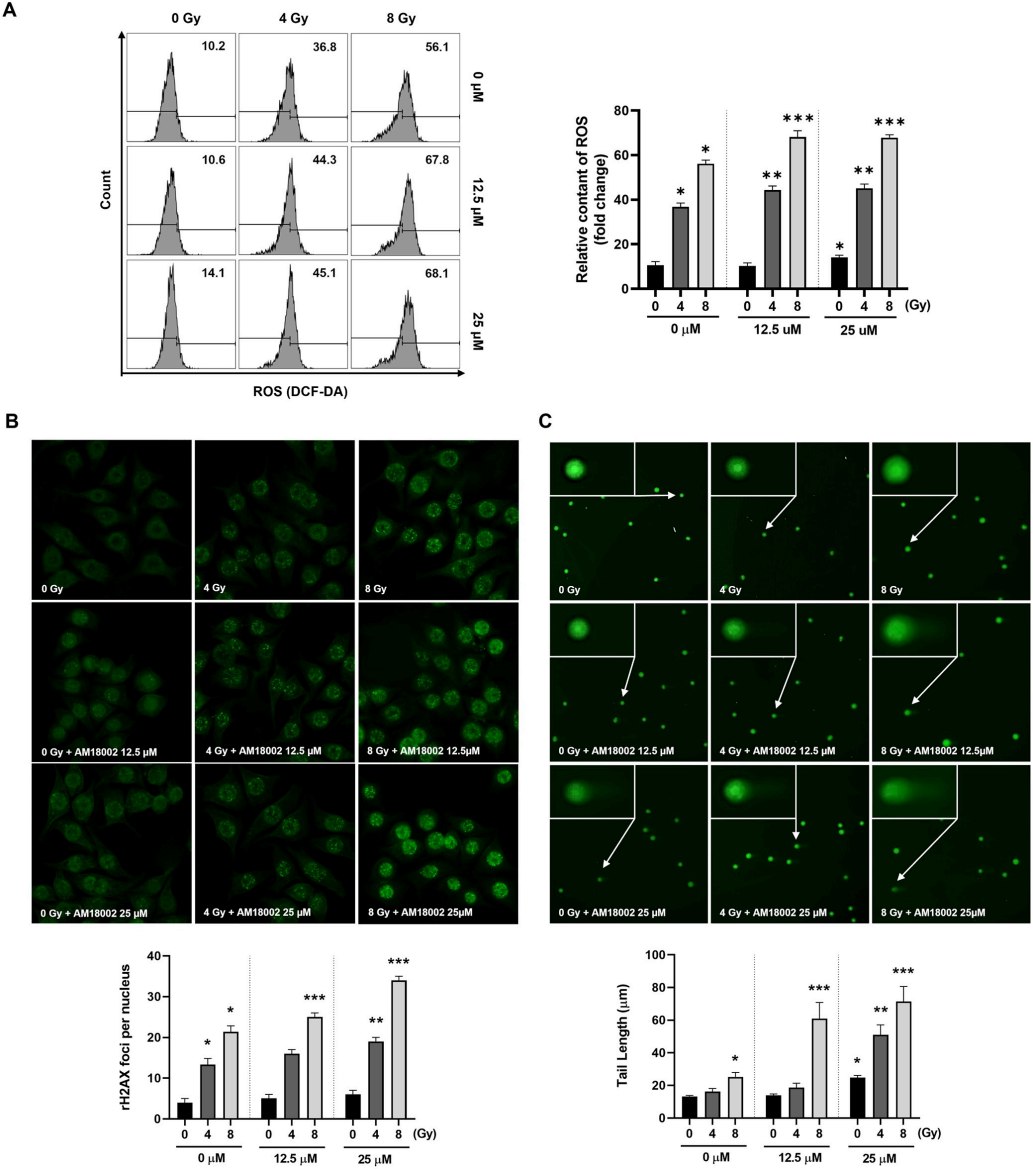
AM-18002 regulates reactive oxygen species responsible for survival signals during the radiation response, promoting apoptosis and significantly increasing DNA damage by irradiation in FM3A cells. (A) FM3A cells were irradiated at 4 or 8 Gy without or with AM-18002 treatment (12.5 or 25 μM). The levels of intracellular reactive oxygen species (ROS) in FM3A cells were determined by measuring the mean fluorescence intensity (MFI) of DCFH-DA (DCF). (B) FM3A cells were immunostained for γ-H2AX foci after treatment with AM-18002 and irradiation. The γ-H2AX foci expression was assessed by fluorescence microscopy. (C) DNA damage was assessed by using an alkaline comet assay. Representative images of the comet assay were taken by fluorescence microscopy. *P < 0.05 indicates a significant difference compared to the respective radiation-only group. The results are presented as the mean ±SD. *P < 0.05 vs. 0 Gy (0 μM), **P < 0.05 vs. 4 Gy (0 μM), ***P < 0.05 vs. 8 Gy (0 μM).

Intracellular ROS are a vital player in cell survival. Therefore, ROS levels in radiation-exposed FM3A cells were measured. We compared the effects of AM-18002 on IR-induced ROS levels in the FM3A cells to determine whether this compound increased the radiosensitivity of these cells by increasing intracellular ROS levels. Flow cytometry revealed that AM-18002 dose-dependently induces ROS generation. The combination of AM-18002 and IR significantly increased ROS levels in the FM3A cells after 4 or 8 Gy irradiation (Fig 3A).

IR induces mitotic arrest directly and indirectly by inducing the production of high ROS levels, thereby leading to DNA damage [34]. The AM-18002-treated cells were irradiated to determine whether ROS accumulation was functionally crucial for AM-18002-induced apoptosis. Then, immunofluorescence was performed to detect γ-H2AX foci, an indicator of radiation-induced DNA double-strand breaks (DSBs). Fig 3B shows the γ-H2AX foci in unirradiated FM3A cells treated with DMSO or AM-18002 alone and FM3A cells subjected to 4 or 8 Gy radiation with or without AM-18002 treatment (12.5 or 25 μM). Without irradiation, AM-18002 produced no γ-H2AX foci. The number of γ-H2AX foci dose-dependently increased in the irradiated FM3A cells. After 24 h of irradiation, AM-18002 (12.5 or 25 μM) combined with 8 Gy irradiation produced more γ-H2AX foci than 8 Gy irradiation alone. The sustained increase in γ-H2AX foci in the FM3A cells after irradiation suggested that AM-18002-mediated radiosensitization involves an increase in DSBs and the inhibition of DNA repair.

Next, to determine whether AM-18002 directly affects DNA damage, a comet assay was conducted. The comet tail length significantly increased in the AM-18002-treated and irradiated FM3A cells compared with the cells subjected to irradiation alone (Fig 3C). Thus, these data suggested that AM-18002 enhances IR-induced DSB generation to promote FM3A cell apoptosis.

### AM-18002 represses tumor-induced MDSCs and STAT3 activation

**Fig 4:**
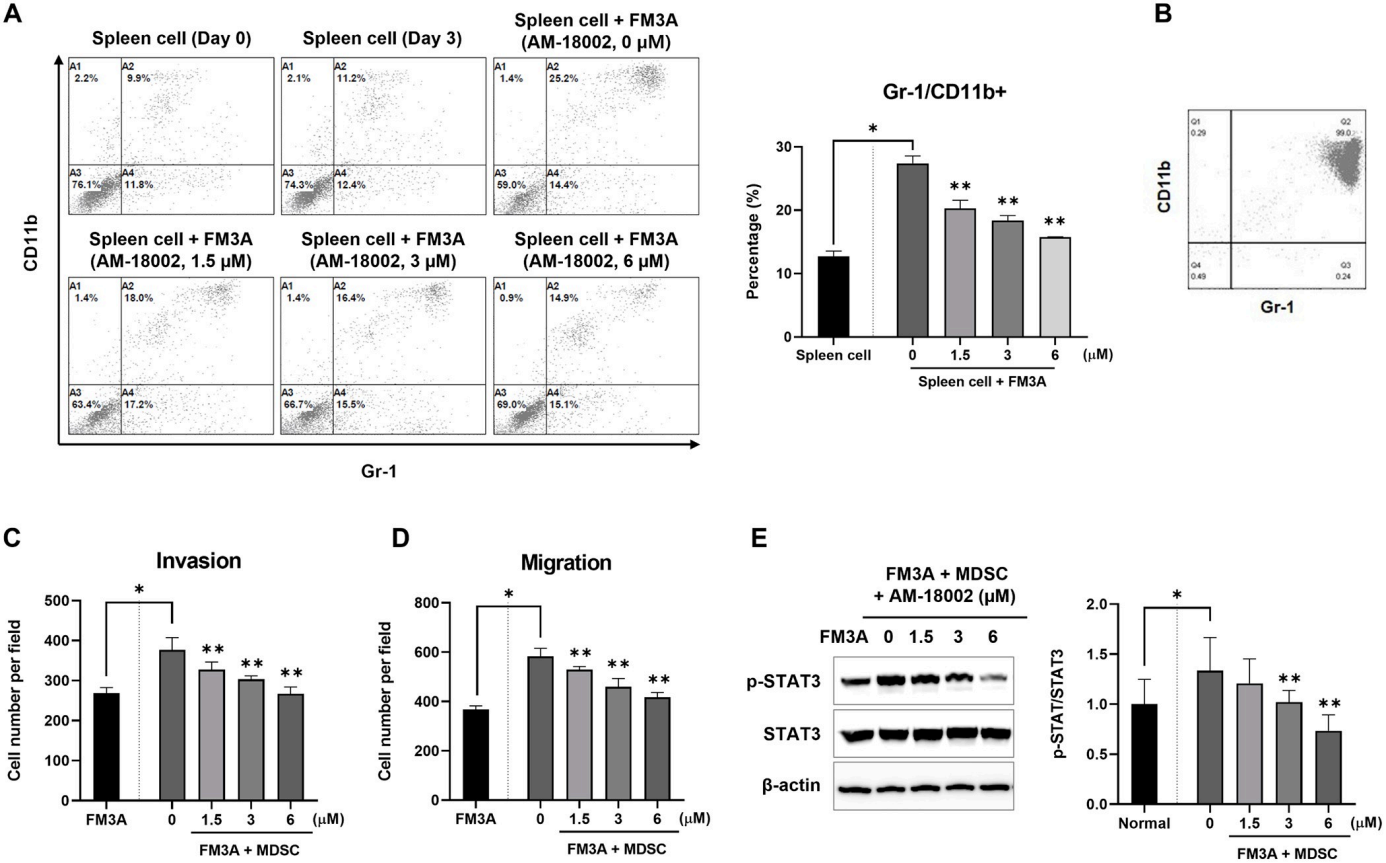
Mouse FM3A breast cancer cells induce MDSC expansion and AM-18002 represses cancer cell-induced MDSC generation. Spleen cells were isolated from FM3A cell tumor-bearing mice and stained with anti-CD11b and anti-Gr-1 antibodies for flow cytometry. Both CD11b and Gr-1 were expressed only by MDSCs. (A) Flow cytometry plots and graphs depicting the percentages of MDSCs among spleen cells under different culture conditions for 72 h. (B) MDSCs were isolated from the spleens of FM3A cell tumor-bearing mice, and the percentage of MDSCs was evaluated again by flow cytometry. (C) and (D) Representative graphs of FM3A cell invasion and migration efficiency after co-culture with MDSCs in the presence of AM-18002. (E) Phospho-STAT3 and STAT3 levels in FM3A cells were determined by Western blotting after removing MDSCs. ß-actin was used as the loading control. The results are presented as the mean ± SD. *P < 0.05 vs. normal (spleen cell or FM3A), **P < 0.05 vs. control (0 μM).

MDSCs are a heterogeneous population of immature myeloid cells comprising granulocytes, macrophages, and dendritic cell precursors that accumulate during chronic inflammation and tumor progression [15, 35–37]. The percentage of MDSCs under co-culture conditions was analyzed through flow cytometry to determine the effects of AM-18002 on FM3A cell-induced MDSC expansion *in vitro*. The MDSC phenotype in the mice was Gr1^+^CD11b^+^, and we therefore analyzed these populations through double-staining. The number of double-positive cells (Gr1^+^CD11b^+^) increased when MDSCs were co-cultured with the FM3A cells, whereas AM-18002 treatment significantly dose-dependently decreased the number of these cells (Fig 4A). The results indicate that the FM3A breast cancer cells promoted MDSC generation and that AM-18002 inhibited this activity.

**Fig 5:**
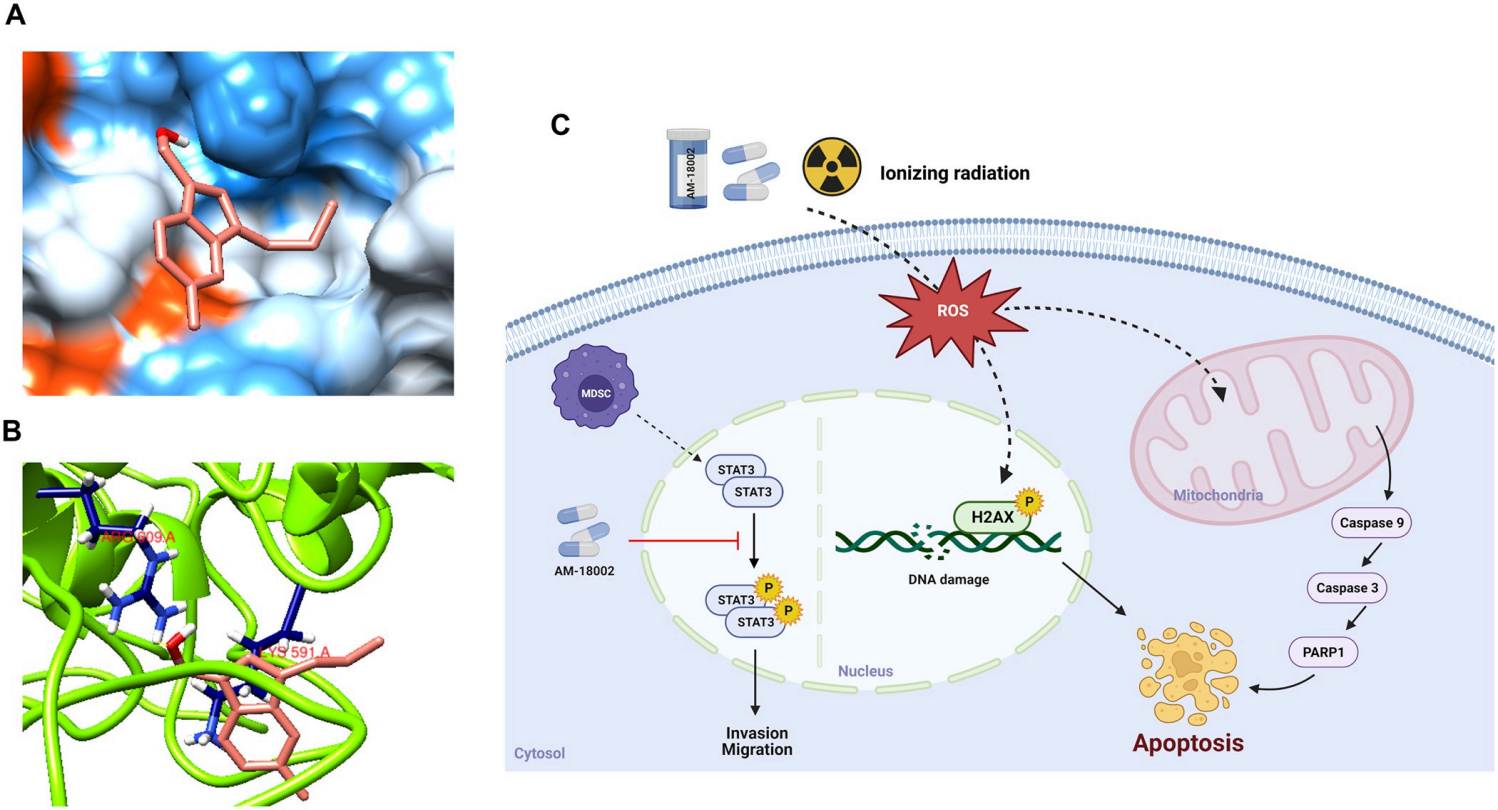
Docked complex of AM-18002 with STAT3. (A) The surface representation of STAT3 displaying deep penetration of AM-18002 into the SH2 domain of STAT3 (PDB:1GB1) (B) Docked conformation of AM-18002 in the SH2 domain of STAT3 (PDB:1BG1). The key amino acid residues are represented as stick structures (color code: navy blue). Hydrogen bonds are shown as yellow lines. (C) Schematic overview of apoptosis induced by AM-18002 via ROS-mediated induction in mouse breast cancer FM3A cells and the signaling pathways that mediate the inhibitory effect of AM-18002 on cancer cell migration and invasion. The image was created using BioRender (www.biorender.com).

In subsequent experiments, MDSCs were isolated from the spleens of the FM3A cell tumor-bearing mice through MACS, and the MDSC abundance in the sorted cell preparation was reconfirmed at 99% (Fig 4B). MDSCs mediate tumor-induced immunosuppressive activities and are directly implicated in promoting tumor angiogenesis, invasion, and metastasis [38]. Therefore, the effects of AM-18002 on MDSC-induced FM3A cell invasion and migration were examined. MDSCs enhanced FM3A cell invasion and migration compared with the untreated control group. This enhancement was significantly dose-dependently prevented by AM-18002 treatment (Figs 4C and 5D). These results indicate that AM-18002 can regulate the MDSC-mediated migration and invasion of FM3A cancer cells.

MDSCs triggered persistent STAT3 activation and increased the invasiveness of breast cancer cells [39]. STAT3 phosphorylation was examined through western blotting to investigate whether AM-18002 affected the activation of STAT3 signaling pathways. FM3A cells co-cultured with MDSCs exhibited STAT3 phosphorylation. However, AM-18002 prevented persistent STAT3 activation (Fig 4E). These data suggest that AM-18002 treatment potently blocks MDSC-mediated STAT3 phosphorylation in FM3A cells.

### Molecular docking against targeted protein

On visualizing the complex (Fig 5A), we further noted that AM-18002 penetrated deep inside the STAT3 cavity and settled well by establishing a network of hydrogen bonding and electrostatic forces with the STAT3 protein. Specifically, AM-18002 used its oxygen atom (hydrogen bond acceptor) of the benzofulvene moiety to form hydrogen bonds with the amine (hydrogen bond donor) functionalities of Arg609 in the STAT3 SH2 domain (Fig 5B). Several anticancer agents targeting Arg609 have been reported [40, 41]. Additionally, hydrophobic interaction and electrostatic forces between the NH_2_ group of Lys591 and the aromatic framework of AM-18002 facilitated the locking of its conformation in the STAT3 binding domain. Overall, the docking results unveiled favorable binding of AM-18002 to the STAT3 protein and supported our experimental results.

## Discussion

RT is among the common approaches for cancer therapy. It can be used alone or combined with chemotherapy and/or surgery [42]. Although RT is among the most effective cancer treatments, many patients exhibit radioresistance [43]. IR combined with other treatment modes may act as a promising strategy against radioresistant cancers. IR can directly or indirectly cause DNA damage. Natural products, including crude extracts, bioactive component-enriched fractions, herb-derived pure compounds, and herbal formulas, prevent and treat cancer [44]. Natural anmindenol A was isolated from the marine-derived bacteria *Streptomyces* sp. [45]. Anmindenol A exerts potent inhibitory activity against inducible nitric oxide synthase without exhibiting any significant cytotoxicity. We here investigated whether a novel derivative of anmindenol A, AM-18002, combined with IR can act synergistically as a potent anticancer agent.

Apoptosis, or programmed cell death, is a highly regulated cell death mechanism involved in many physiological processes, such as development, elimination of damaged cells, and the immune response [46–54]. First, the effect of AM-18002 on radiation-induced apoptosis was determined through flow cytometry. Compared with irradiation alone, AM-18002 combined with IR synergistically induced FM3A cell apoptosis (Fig 2A). Mitochondrial permeability transition is a key event in apoptosis. Therefore, the mitochondrial function was evaluated by assessing the mitochondrial membrane potential. AM-18002 significantly increased the degree of green fluorescence, thereby indicating that the AM-18002 and IR combination decreased the mitochondrial membrane potential (Fig 2C). Defects in caspase cascade activation are known to induce tumor progression and metastasis, whereas the activation of this cascade by compounds that sensitize tumor cells to irradiation-induced apoptosis reduced tumor sizes and controlled cancer development [55, 56]. Caspase 3 serves as an executor of apoptosis, and many radiosensitizers, including AM-18002, target the activation of the caspase cascade (Fig 2E). Colonies formed by AM-18002-treated cells were significantly smaller than those formed by the irradiated cells (Fig 2F). The aforementioned results indicated that AM-18002 induces apoptosis, thereby increasing the radiosensitivity of FM3A cells.

Irradiation- and chemotherapeutic agent-mediated induction of apoptosis is a known strategy for killing tumor cells. Irradiation leads to the generation of ROS and free radicals, thereby inducing DNA damage. DSBs are the most abundant and toxic irradiation-induced DNA damage [57]. The AM-18002 and IR combination significantly increased ROS levels and more effectively induced DNA damage, as observed through the upregulated expression of γ-H2AX foci and increased comet tail length in the cells treated with the aforementioned combination therapy compared with irradiation treatment alone (Fig 3). These results suggested the utility of AM-18002 as a sensitizer for FM3A cell irradiation.

MDSCs, which can exert immunosuppressive functions through multiple pathways and mechanisms, are crucial players in tumor evasion [35]. ROS generation and interleukin (IL)-6-triggered STAT3 activation regulates MDSC expansion in breast cancer [58]. Yu et al. observed STAT3 activation in various cancers and reported that this activation might control cancer progression and metastasis [17, 59–63]. MDSCs strongly induce STAT3 phosphorylation in breast cancer cells co-cultured with MDSCs [64]. MDSCs were expanded by co-culture with the FM3A cells and were significantly inhibited by AM-18002 (Fig 4). MDSC-enhanced invasion, migration, and STAT3 phosphorylation decreased dose-dependently after AM-18002 treatment. In Fig 5, the docking results revealed the favorable binding of AM-18002 to the STAT3 protein and supported our experimental results. Altogether, these results suggest that AM-18002 is an effective STAT3 inhibitor and MDSC depletion inhibited the migration and invasion of breast cancer cells.

In this study, the anmindenol A derivative AM-18002 served as a radiosensitizer and anticancer agent in FM3A mouse breast cancer cells. The AM-18002 and IR combination synergistically induced cancer cell death by increasing ROS generation, and AM-18002 increased the radiosensitivity of FM3A cells. AM-18002 exerted its anticancer effect by inhibiting MDSC expansion and decreasing the invasion and migration of cancer cells, which was related to the suppression of STAT3 phosphorylation (Fig 5C). Therefore AM-18002 can be a promising therapeutic strategy against breast cancer.

## Supporting information

S1 Raw images (PDF)

S1 File HPLC chromatograms of AM-18002. (PDF)

## References

[1] Wessler S, Aberger F, Hartmann T. The sound of tumor cell-microenvironment communication-composed by the cancer cluster salzburg research network. Cell Commun Signa.l 2017;15(1):1–2. doi: 10.1186/s12964-017-0176-z . 28577565 PMC5457625

[2] Wang Y, Liang R, Fang F. Applications of nanomaterials in radiotherapy for malignant tumors. J Nanosci Nanotechnol. 2005;15(8):5487–500. doi: 10.1166/jnn.2015.10617 . 26369108

[3] Lim ZZJ, Li JE, Ng CT, Yung LYL, Bay BH. Gold nanoparticles in cancer therapy. Acta Pharmacol Sin. 2011;32(8):983–990. doi: 10.1038/aps.2011.82 . 21743485 PMC4002534

[4] Haume K, Rosa S, Grellet S, Smialek MA, Butterworth KT, Solov’yov AV, et al. Gold nanoparticles for cancer radiotherapy: a review. Cancer Nanotechnol. 2016;7(1):1–20. doi: 10.1186/s12645-016-0021-x . 27867425 PMC5095165

[5] Malik A, Sultana M, Qazi A, Qazi MH, Parveen G, Waquar S, et al. Role of natural radiosensitizers and cancer cell radioresistance: an update. Anal Cell Pathol. 2016:6146595. doi: 10.1155/2016/6146595 . 26998418 PMC4779816

[6] Raviraj J, Bokkasam VK, Kumar VS, Reddy US, Suman V. Radiosensitizers, radioprotectors, and radiation mitigators. Indian J Dent Res. 2014:25(1): 83–90. doi: 10.4103/0970-9290.131142 . 24748306

[7] McBride HM, Neuspiel M, Wasiak S. Mitochondria: more than just a powerhouse. Current Biology. 2006;16(14):R551–560. doi: 10.1016/j.cub.2006.06.054 . 16860735

[8] Allen J, Romay-Tallon R, Brymer KJ, Caruncho HJ, Kalynchuk LE. Mitochondria and mood: mitochondrial dysfunction as a key player in the manifestation of depression. Frontiers in Neurosci. 2018;12:386. doi: 10.3389/fnins.2018.00386 . 29928190 PMC5997778

[9] Petit PX, Susin SA, Zamzami N, Mignotte B, Kroemer G. Mitochondria and programmed cell death: back to the future. FEBS Lett. 1996;396(1):7–13. doi: 10.1016/0014-5793(96)00988-x . 8906857

[10] Gottieb RA. Mitochondria and apoptosis. Biol Signals. 2001;10(3–4):147–161. doi: 10.1159/000046884 . 11351125

[11] Henkart PA, Grinstein S. Apoptosis: mitochondria resurrected?. J Exp Med. 1996;183(4):1293–1295. doi: 10.1084/jem.183.4.1293 . 8666886 PMC2192486

[12] Pouget JP, Frelon S, Ravanat JL, Testard I, Odin F, Cadet J. Formation of modified DNA bases in cells exposed either to gamma radiation or to high-LET particles. Radiat Res. 2002;157(5):589–595. doi: 10.1667/0033-7587(2002)157[0589:fomdbi]2.0.co;2 . 11966325

[13] Sachs RK, Chen PL, Hahnfeldt PJ, Hlatky LR. DNA damage caused by ionizing radiation. Math Biosci. 1992;112(2):271–303. doi: 10.1016/0025-5564(92)90028-u . 1490054

[14] Stewart TJ, Abrams SI. How tumours escape mass destruction. Oncogene. 2008;27(45):5894–5903. doi: 10.1038/onc.2008.268 . 18836470

[15] Gabrilovich DI, Ostrand-Rosenberg S, Bronte V. Coordinated regulation of myeloid cells by tumours. Nat Rev Immunol. 2012;12(4):253–268. doi: 10.1038/nri3175 . 22437938 PMC3587148

[16] Mukthavaram R, Ouyang X, Saklecha R, Jiang P, Nomura N, Pingle SC, et al. Effect of the JAK2/STAT3 inhibitor SAR317461 on human glioblastoma tumorspheres. J Transl Med. 2015;18:269–279. doi: 10.1186/s12967-015-0627-5 . 26283544 PMC4539675

[17] Ma JH, Qin L, Li X. Role of STAT3 signaling pathway in breast cancer. Cell Commun Signal. 2020;18(1):33. doi: 10.1186/s12964-020-0527-z . 32111215 PMC7048131

[18] Yin T, Zhao ZB, Guo J, Wang T, Yang JB, Wang C, et al. Aurora A inhibition eliminates myeloid cell-mediated immunosuppression and enhances the efficacy of anti-PD-L1 therapy in breast cancer. Cancer Res. 2019;79(13):3431–3444. doi: 10.1158/0008-5472.CAN-18-3397 . 30902796

[19] Jo J, Jeong M, Ahn JS, Akter J, Kim HS, Suh YG, et al. Total synthesis of Anmindenol A and its application to the design, synthesis, and biological evaluation of derivatives thereof. J Org Chem. 2019;84(17):10953–10961. doi: 10.1021/acs.joc.9b01564 . 31357857

[20] Razzaghi-Asl N, Mirzayi S, Mahnam K, Sepehri S. Identification of COX-2 inhibitors via structure-based virtual screening and molecular dynamics simulation. J Mol Graph Model. 2018;83:138–152. doi: 10.1016/j.jmgm.2018.05.010 . 29936228

[21] Pires DE, Blundell TL, Ascher DB. pkCSM: predicting small-molecule pharmacokinetic and toxicity properties using graph-based signatures. J Med Chem. 2015;58(9):4066–4072. doi: 10.1021/acs.jmedchem.5b00104 . 25860834 PMC4434528

[22] Ahn SY, Jo MS, Lee D, Baek SE, Baek J, Yu JS, et al. Dual effects of isoflavonoids from Pueraria lobata roots on estrogenic activity and anti-proliferation of MCF-7 human breast carcinoma cells. Bioorg Chem. 2019;83:135–144. doi: 10.1016/j.bioorg.2018.10.017 . 30352359

[23] Forli S, Huey R, Pique ME, Sanner MF, Goodsell DS, Olson AJ. Computational protein-ligand docking and virtual drug screening with the AutoDock suite. Nat Protoc. 2016;11(5):905–919. doi: 10.1038/nprot.2016.051 . 27077332 PMC4868550

[24] Pettersen EF, Goddard TD, Huang CC, Couch GS, Greenblatt DM, Meng EC, et al. UCSF Chimera—a visualization system for exploratory research and analysis. J Comput Chem. 2004;25(13):1605–1612. doi: 10.1002/jcc.20084 . 15264254

[25] Martin YC. A bioavailability score. J Med Chem. 2005;48(9):3164–3170. doi: 10.1021/jm0492002 . 15857122

[26] Veber DF, Johnson SR, Cheng HY, Smith BR, Ward KW, Kopple KD. Molecular properties that influence the oral bioavailability of drug candidates. J Med Chem. 2002;45(12):2615–2623. doi: 10.1021/jm020017n . 12036371

[27] Toolabi M, Moghimi S, Bakhshaiesh TO, Salarinejad S, Aghcheli A, Hasanvand Z, et al. 6-Cinnamoyl-4-arylaminothienopyrimidines as highly potent cytotoxic agents: Design, synthesis and structure-activity relationship studies. Eur J Med Chem. 2020;185:111786. doi: 10.1016/j.ejmech.2019.111786 . 31671308

[28] Hann MM, Oprea TI. Pursuing the leadlikeness concept in pharmaceutical research. Curr Opin Chem Biol. 2004;8(3):255–263. doi: 10.1016/j.cbpa.2004.04.003 15183323

[29] Lipinski CA, Lombardo F, Dominy BW, Feeney PJ. Experimental and computational approaches to estimate solubility and permeability in drug discovery and development settings. Adv Drug Deliv Rev. 2001;46(1–3):3–26. doi: 10.1016/s0169-409x(00)00129-0 11259830

[30] Baell J, Walters MA. Chemistry: Chemical con artists foil drug discovery. Nature. 2014;513(7519):481–483. doi: 10.1038/513481a 25254460

[31] Shamsuddin T, Hosen MA, Alam M S, Emran TB, Kawsar SM A. Uridine derivatives: Antifungal, PASS outcomes, ADME/T, drug-likeliness, molecular docking and binding energy calculations. Med Sci Int Med J. 2021;10:1373–86.

[32] Fulda S. Targeting apoptosis for anticancer therapy. Semin Cancer Biol. 2015;31:84–88. doi: 10.1016/j.semcancer.2014.05.002 . 24859747

[33] Chen T, Wong YS. Selenocystine induces caspase-independent apoptosis in MCF-7 human breast carcinoma cells with involvement of p53 phosphorylation and reactive oxygen species generation. Int J Biochem Cell Biol. 2009;41(3):666–676. doi: 10.1016/j.biocel.2008.07.014 . 18718551

[34] Perillo B, Di Donato M, Pezone A, Di Zazzo E, Giovannelli P, Galasso G, et al. ROS in cancer therapy: the bright side of the moon. Exp Mol Med. 2020;52(2):192–203. doi: 10.1038/s12276-020-0384-2 . 32060354 PMC7062874

[35] Kumar V, Patel S, Tcyganov E, Gabrilovich DI. The nature of myeloid-derived suppressor cells in the tumor microenvironment. Trends Immunol. 2016;37(3):208–220. doi: 10.1016/j.it.2016.01.004 . 26858199 PMC4775398

[36] Parker KH, Beury DW, Ostrand-Rosenberg S. Myeloid-derived suppressor cells: critical cells driving immune suppression in the tumor microenvironment. Adv Cancer Res. 2015;128:95–139. doi: 10.1016/bs.acr.2015.04.002 . 26216631 PMC4662416

[37] Meirow Y, Kanterman J, Baniyash M. Paving the road to tumor development and spreading: myeloid-derived suppressor cells are ruling the fate. Front Immunol. 2015;12:523. doi: 10.3389/fimmu.2015.00523 . 26528286 PMC4601280

[38] Gabrilovich DI, Nagaraj S. Myeloid-derived suppressor cells as regulators of the immune system. Nat Rev Immunol. 2009;9(3):162–174. doi: 10.1038/nri2506 . 19197294 PMC2828349

[39] Oh K, Lee OY, Shon SY, Nam O, Ryu PM, Seo MW, et al. A mutual activation loop between breast cancer cells and myeloid-derived suppressor cells facilitates spontaneous metastasis through IL-6 trans-signaling in a murine model. Breast Cancer Research. 2013;15(5):R79. doi: 10.1186/bcr3473 . 24021059 PMC3979084

[40] Yu W, Xiao H, Lin J, Li C. Discovery of novel STAT3 small molecule inhibitors via in silico site-directed fragment-based drug design. J Med Chem. 2013;56(11):4402–4412. doi: 10.1021/jm400080c . 23651330

[41] Bhasin D, Cisek K, Pandharkar T, Regan N, Li C, Pandit B, et al. Design, synthesis, and studies of small molecule STAT3 inhibitors. Bioorg Med Chem Lett. 2008;18(1):391–395. doi: 10.1016/j.bmcl.2007.10.031 . 18006313

[42] Krause M, Yaromina A, Eicheler W, Koch U, Baumann M. Cancer stem cells. targets and potential biomarkers for radiotherapy. Clin Cancer Res. 2011;17(23):7224–7229. doi: 10.1158/1078-0432.CCR-10-2639 . 21976536

[43] Begg AC, Stewart FA, Vens C. Strategies to improve radiotherapy with targeted drugs. Nat Rev Cancer. 2011;11(4):239–253. doi: 10.1038/nrc3007 . 21430696

[44] Sanders K, Moran Z, Shi Z, Paul R, Greenlee H. Natural products for cancer prevention: clinical update 2016. Semin Oncol Nurs. 2016;32(3):215–240. doi: 10.1016/j.soncn.2016.06.001 . 27539278

[45] Lee J, Kim H, Lee TG, Yang I, Won DH, Choi H, et al. Anmindenols A and B, inducible nitric oxide synthase inhibitors from a marine-derived streptomyces sp. J Nat Prod. 2014;77(6):1528–1531. doi: 10.1021/np500285a . 24878306

[46] Hengartner M. The biochemistry of apoptosis. Nature. 2000;407(6805):770–776. doi: 10.1038/35037710 . 11048727

[47] Jacobson M, Weil M, Raff M. Programmed cell death in animal development. Cell. 1997;88(3):347–354. doi: 10.1016/s0092-8674(00)81873-5 . 9039261

[48] Leist M, Jäättelä M. Four deaths and a funeral: from caspases to alternative mechanisms. Nat Rev Mol Cell Biol. 2001;2(8):589–598. doi: 10.1038/35085008 . 11483992

[49] Oppenheim R. Cell death during development of the nervous system. Ann Rev Neurosci. 1991;14:453–501. doi: 10.1146/annurev.ne.14.030191.002321 . 2031577

[50] Raff M. Cell suicide for beginners. Nature. 1998;396(6707):119–122. doi: 10.1038/24055 . 9823889

[51] Lockshin RA, Zakeri Z. Programmed cell death and apoptosis: origins of the theory. Nat Rev Mol Cell Biol. 2001;2(7):545–550. doi: 10.1038/35080097 . 11433369

[52] Zuzarte-Luis V, Hurle JM. Programmed cell death in the developing limb. Int J Dev Biol. 2002;46(7):871–876. . 12455623

[53] Hutchins JB, Barger SW. Why neurons die: cell death in the nervous system. Anat Rec. 1998;253(3):79–90. doi: 10.1002/(SICI)1097-0185(199806)253:3&lt;79::AID-AR4&gt;3.0.CO;2-9 . 9700393

[54] Meier P, Finch A, Evan G. Apoptosis in development. Nature. 2000;407(6805):796–801. doi: 10.1038/35037734 . 11048731

[55] Ju GZ, Shen B, Sun SL, Yan FQ, Fu SB. Effect of X-rays on expression of caspase-3 and p53 in EL-4 cells and its biological implications. Biomed Environ Sci. 2007;20(6):456–459. . 18348402

[56] Hosokawa Y, Sakakura Y, Tanaka L, Okumura K, Yajima T, Kaneko M. Radiationinduced apoptosis is independent of caspase-8 but dependent on cytochrome c and the caspase-9 cascade in human leukemia HL60 cells. J Radiat Res. 2005;46(3):293–303. doi: 10.1269/jrr.46.293 . 16210785

[57] Roos WP, Kaina B. DNA damage-induced cell death. from specific DNA lesions to the DNA damage response and apoptosis. Cancer Lett. 2013;332(2):237–248. doi: 10.1016/j.canlet.2012.01.007 . 22261329

[58] Narayanan PD, Nandabalan SK, Baddireddi LS. Role of STAT3 phosphorylation in ethanol-mediated proliferation of breast cancer cells. J Breast Cancer. 2016;19(2):122–132. doi: 10.4048/jbc.2016.19.2.122 . 27382387 PMC4929252

[59] Yu H, Kortylewski M, Pardoll D. Crosstalk between cancer and immune cells: role of STAT3 in the tumour microenvironment. Nat Rev Immunol. 2007;7(1):41–51. doi: 10.1038/nri1995 . 17186030

[60] Sansone P, Storci G, Tavolari S, Guarnieri T, Giovannini C, Taffurelli M, et al. IL-6 triggers malignant features in mammospheres from human ductal breast carcinoma and normal mammary gland. J Clin Invest. 2007;117(12):3988–4002. doi: 10.1172/JCI32533 . 18060036 PMC2096439

[61] Gao SP, Mark KG, Leslie K, Pao W, Motoi N, Gerald WL, et al. Mutations in the EGFR kinase domain mediate STAT3 activation via IL-6 production in human lung adenocarcinomas. J Clin Invest. 2007;117(12):3846–3856. doi: 10.1172/JCI31871 . 18060032 PMC2096430

[62] Grivennikov S, Karin E, Terzic J, Mucida D, Yu GY, Vallabhapurapu S, et al. IL-6 and Stat3 are required for survival of intestinal epithelial cells and development of colitis-associated cancer. Cancer Cell. 2009;15(2):103–113. doi: 10.1016/j.ccr.2009.01.001 . 19185845 PMC2667107

[63] Marotta LL, Almendro V, Marusyk A, Shipitsin M, Schemme J, Walker SR, et al. The JAK2/STAT3 signaling pathway is required for growth of CD44+CD24- stem cell-like breast cancer cells in human tumors. J Clin Invest. 2011;121(7):2723–2735. doi: 10.1172/JCI44745 . 21633165 PMC3223826

[64] Peng D, Tanikawa T, Li W, Zhao L, Vatan L, Szeliga W, et al. Myeloid-derived suppressor cells endow stemlike qualities to breast cancer cells through IL6/STAT3 and NO/NOTCH cross-talk signaling. Cancer Res. 2016;76(11):3156–3165. doi: 10.1158/0008-5472.CAN-15-2528 . 27197152 PMC4891237

