# Supplementary figures and images for: AM-18002, a derivative of natural anmindenol A, enhances radiosensitivity in mouse breast cancer cells
Source: PLoS One. 2024 Apr 16;19(4):e0296989. doi: 10.1371/journal.pone.0296989 (PMC11020960; doi:10.1371/journal.pone.0296989)

Figure. 2E

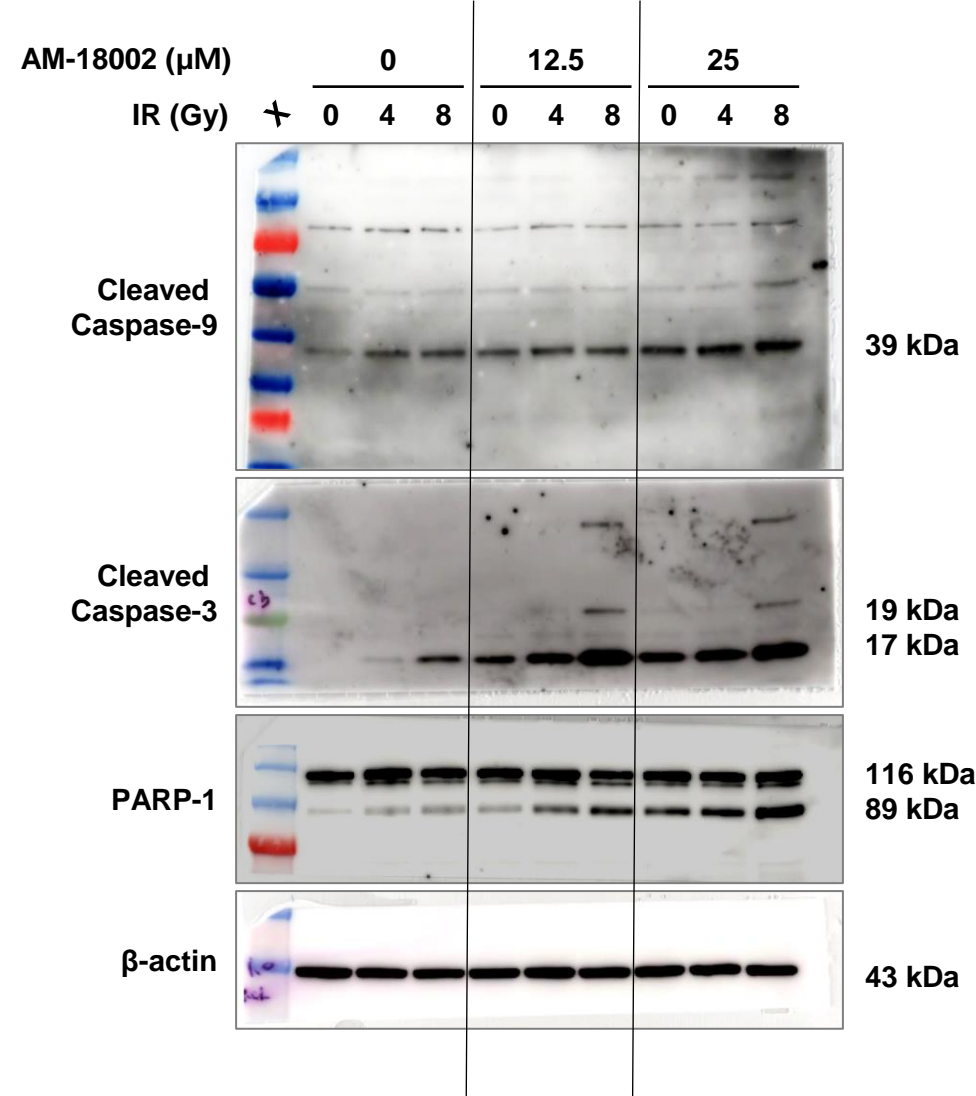

Figure. 4E

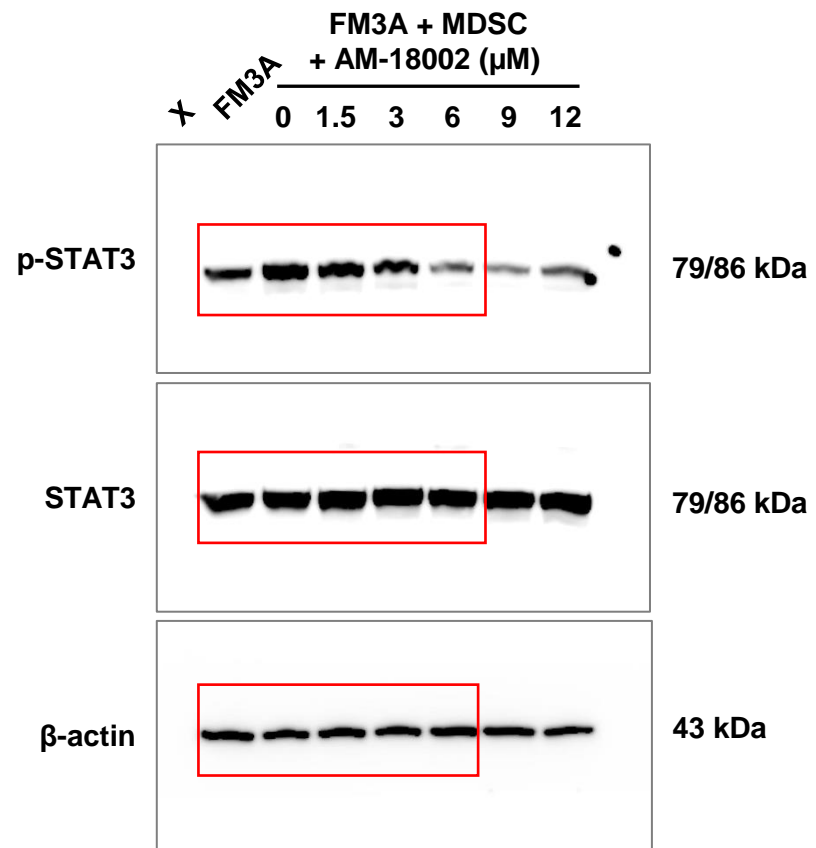

Supplement: S1 Raw images — (PDF) [file pone.0296989.s001.pdf]
