# Supplementary material for: AM-18002, a derivative of natural anmindenol A, enhances radiosensitivity in mouse breast cancer cells
Source: PLoS One. 2024 Apr 16;19(4):e0296989. doi: 10.1371/journal.pone.0296989 (PMC11020960; doi:10.1371/journal.pone.0296989)
Supplement: S1 File — (PDF) [file pone.0296989.s002.pdf]

HPLC chromatograms of AM-18002

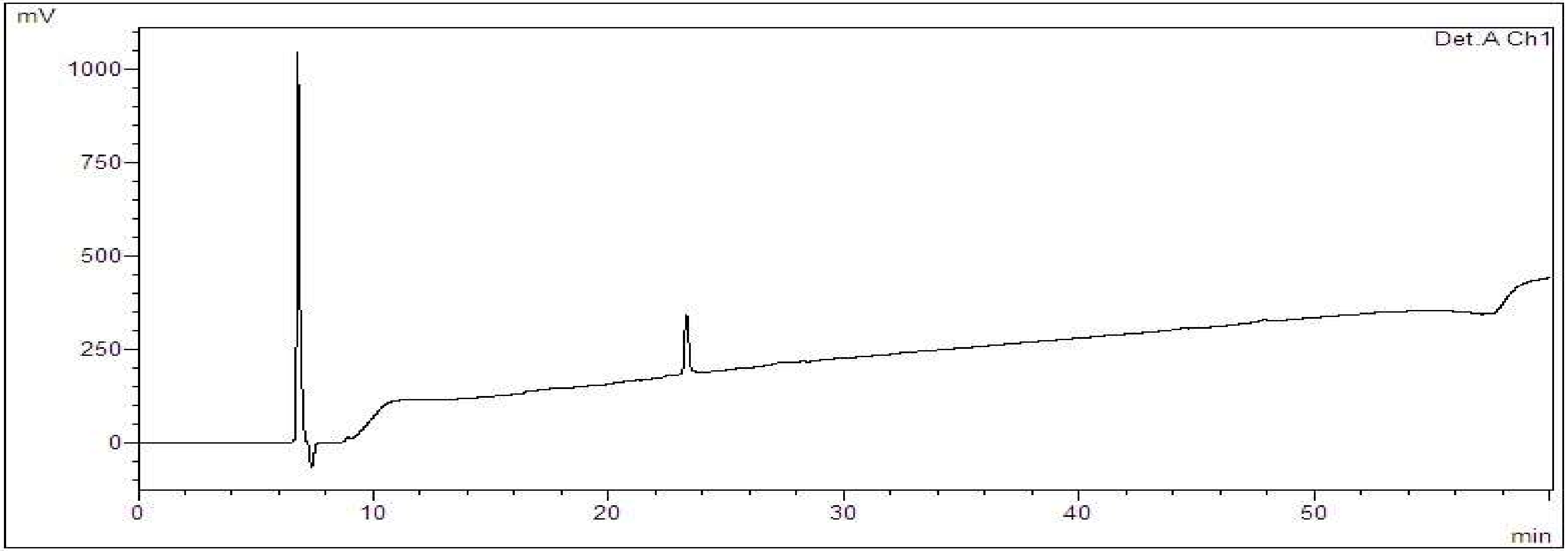

S2. The HPLC analysis for the purity of AM-18002 (detection wavelength was set as 220 nm)
